# Mendelian randomisation analysis of red cell distribution width in pulmonary arterial hypertension

**DOI:** 10.1183/13993003.01486-2019

**Published:** 2020-02-13

**Authors:** Anna Ulrich, John Wharton, Timothy E. Thayer, Emilia M. Swietlik, Tufik R. Assad, Ankit A. Desai, Stefan Gräf, Lars Harbaum, Marc Humbert, Nicholas W. Morrell, William C. Nichols, Florent Soubrier, Laura Southgate, David-Alexandre Trégouët, Richard C. Trembath, Evan L. Brittain, Martin R. Wilkins, Inga Prokopenko, Christopher J. Rhodes

**Affiliations:** 1 National Heart and Lung Institute, Hammersmith Campus, Imperial College London, London, UK; 2 Vanderbilt University Medical Center, Division of Cardiovascular Medicine, Nashville, TN, USA; 3 Dept of Medicine, University of Cambridge, Cambridge, UK; 4 Pulmonary Vascular Disease Unit, Royal Papworth Hospital NHS Foundation Trust, Cambridge, UK; 5 Williamson Country Medical Center, Franklin, TN, USA; 6 Dept of Medicine, Indiana University, Indianapolis, IN, USA; 7 NIHR BioResource – Rare Diseases, Cambridge, UK; 8 Dept of Haematology, University of Cambridge, Cambridge, UK; 9 Université Paris-Sud, Faculté de Médecine, Université Paris-Saclay, Paris, France; 10 AP-HP, Service de Pneumologie, Centre de référence de l'hypertension pulmonaire, Hôpital Bicêtre, Le Kremlin-Bicêtre, France; 11 INSERM UMR_S 999, Hôpital Marie Lannelongue, Le Plessis Robinson, France; 12 Division of Human Genetics, Cincinnati Children's Hospital Medical Center, Dept of Pediatrics, University of Cincinnati College of Medicine, Cincinnati, OH, USA; 13 Sorbonne Universités, UPMC Univ. Paris 06, Institut National pour la Santé et la Recherche Médicale (INSERM), Unité Mixte de Recherche en Santé (UMR_S) 1166, Paris, France; 14 Molecular and Clinical Sciences Research Institute, St George's University of London, London, UK; 15 INSERM UMR_S 1219, Bordeaux Population Health Research Center, University of Bordeaux, Bordeaux, France; 16 Division of Genetics and Molecular Medicine, King's College London, London, UK; 17 Vanderbilt Translational and Clinical Cardiovascular Research Center, Nashville, TN, USA; 18 Dept of Clinical and Experimental Medicine, University of Surrey, Guildford, UK; 19 Dept of Metabolism, Digestion and Reproduction, Imperial College London, London, UK; 20 Members listed in the supplementary material; 21 These authors contributed equally

## Abstract

Pulmonary arterial hypertension (PAH) is a rare disease that leads to premature death from right heart failure. It is strongly associated with elevated red cell distribution width (RDW), a correlate of several iron status biomarkers. High RDW values can signal early-stage iron deficiency or iron deficiency anaemia. This study investigated whether elevated RDW is causally associated with PAH.

A two-sample Mendelian randomisation (MR) approach was applied to investigate whether genetic predisposition to higher levels of RDW increases the odds of developing PAH. Primary and secondary MR analyses were performed using all available genome-wide significant RDW variants (n=179) and five genome-wide significant RDW variants that act *via* systemic iron status, respectively.

We confirmed the observed association between RDW and PAH (OR 1.90, 95% CI 1.80–2.01) in a multicentre case–control study (cases n=642, disease controls n=15 889). The primary MR analysis was adequately powered to detect a causal effect (odds ratio) between 1.25 and 1.52 or greater based on estimates reported in the RDW genome-wide association study or from our own data. There was no evidence for a causal association between RDW and PAH in either the primary (OR_causal_ 1.07, 95% CI 0.92–1.24) or the secondary (OR_causal_ 1.09, 95% CI 0.77–1.54) MR analysis.

The results suggest that at least some of the observed association of RDW with PAH is secondary to disease progression. Results of iron therapeutic trials in PAH should be interpreted with caution, as any improvements observed may not be mechanistically linked to the development of PAH.

## Abstract 2

**Mendelian randomisation using genetic data from the largest-to-date PAH cohort does not support red cell distribution width or iron deficiency as a cause of PAH, which is important when interpreting iron replacement trials in this condition** http://bit.ly/2PPaa88

## Introduction

Pulmonary arterial hypertension (PAH) is a rare disease with an estimated prevalence of 7–26 cases per million in the developed world [1]. It is characterised by increased pulmonary vascular resistance due to vasoconstriction and structural remodelling of pulmonary arterioles, leading to right ventricular hypertrophy and end-stage right heart failure [2]. Despite increased awareness and new therapeutic options, annual mortality remains ∼10% [1]. Approximately 70–80% of heritable PAH and 10–20% of idiopathic PAH patients are known to harbour mutations in the bone morphogenetic protein type II receptor (*BMPR2*) gene [3]. A recent large study of >1000 PAH patients confirmed the prevalence of causal mutations in *BMPR2*, as well as in five other established genes (*TBX4*, *ACVRL1*, *ENG*, *SMAD9* and *KCNK3*), and identified PAH-associated mutations in four new genes (*ATP13A3*, *SOX17*, *AQP1* and *GDF2*), altogether accounting for 23.5% of the cases studied [4]. The rare mutations in all these genes are inherited in an autosomal dominant manner and exhibit reduced penetrance, indicating that other genetic, epigenetic and/or environmental factors influence the development of PAH.

We and others have demonstrated that one factor strongly correlated with survival in PAH is red cell distribution width (RDW) [5, 6]. A recent hypothesis-free phenome-wide analysis indicated PAH, among several disease descriptors, as the most strongly associated with RDW (OR 2.0, 95% CI 1.75–2.4 per % increase in RDW) in a hospital population [7]. RDW (a measure of red blood cell (RBC) size variability in an individual) is part of the full blood count in standard hospital practice and readily available as a biomarker. RDW correlates with iron status biomarkers and high values can signal early stage iron deficiency or iron deficiency anaemia [8]. RDW increases with decreasing iron as available body iron stores fail to meet the iron demand of RBC synthesis, resulting in RBCs of varied size.

Iron deficiency is commonly observed in PAH patients and is under investigation as a therapeutic target [9–14]. An observed correlation between two traits does not necessarily imply that interventions on one trait will change the other and there are numerous examples where false-positive associations have led to unsuccessful randomised controlled trials [15, 16]. Clinical trials are expensive and time-consuming, and recruitment can be challenging, especially in rare diseases. With the growing availability of genetic data in large disease-focused and population-based studies, testing causal relationships between traits of interest has become possible by harnessing the naturally occurring genetic variation in the population. The collection of methods used to test causal relationships using genetic variants is called Mendelian randomisation (MR) [17, 18]. MR has been used successfully to help prioritise intervention and drug targets and to identify causal factors for several diseases [19–23]. In general, candidate drugs with genetic evidence for effectiveness are more successful in drug trials compared to those without such genetic support [24].

It remains to be investigated whether elevated RDW is largely a consequence of PAH or plays a causal role in the condition. The common genetic variation determining RDW levels has been defined in very large population studies [25], with more power than equivalent studies of iron status. This makes RDW a strong candidate for MR analysis and our primary aim was to dissect the epidemiological association between RDW and PAH by assessing causality using MR. We refined the estimate of association between RDW and PAH using biomarker data in 642 PAH cases and >15 000 controls with common diseases from the Imperial College Pulmonary Hypertension Biobank, the UK PAH cohort and the Vanderbilt University Medical Centre (VUMC) and applied MR to investigate whether being genetically predisposed to higher levels of RDW increases the odds of developing PAH.

## Methods

### Definition of PAH

PAH was defined by internationally agreed criteria [26], specifically mean pulmonary artery pressure >25 mmHg, pulmonary vascular resistance >3 Woods units and pulmonary capillary wedge pressure <15 mmHg. Patients with concurrent diseases known to cause pulmonary hypertension were excluded, such that all were considered to have idiopathic, heritable or anorexigen-induced PAH.

### Data: genetic and phenotype data in contributing studies

For our analyses we used both individual-level and summary-level data. Individual-level data including clinical laboratory RDW were available for 524 PAH patients from the Imperial College Pulmonary Hypertension Biobank and the multicentre UK PAH cohort, a study that facilitates collaboration and the sharing of data and samples between the specialist pulmonary hypertension centres in the UK (www.ipahcohort.com).

In addition, the hospital population-based VUMC study provided longitudinal clinical laboratory RDW measurements, detailed clinical diagnoses and genome-wide genotype array data (genotyping platform: Illumina MEGAex) for an additional 118 PAH patients and 15 889 common disease controls (supplementary table S1). VUMC hosts a collection of electronic medical records linked to genetic data derived from blood collected during routine clinical assessment in outpatient clinics where all patients are shown the consent form during check-in [27] (https://victr.vanderbilt.edu/pub/biovu/) The exclusion criteria (supplementary figure S1) and the imputation of genotype array data are described in the supplementary methods.

Summary-level data for both RDW and PAH susceptibility were used in the MR analyses to maximise the sample size and therefore the statistical power. Genetic instruments serving as a proxy for RDW were obtained from the largest-to-date (>170 000 individuals) genome-wide association study (GWAS) on haematological traits (here referred to as the “RDW GWAS”) in a population-based study by Astle *et al*. [25].

A large proportion of the idiopathic and familial PAH cases from the UK PAH cohort were whole-genome sequenced as part of the UK National Institute for Health Research BioResource (NIHRBR) [4, 28] study. For genetic effects on PAH susceptibility we used a recent study published by our group and others investigating, in the largest-to-date GWAS, the effects of common genetic variation on PAH risk (“PAH GWAS”) involving 11 744 individuals, of which 2085 were PAH cases. The results of the PAH GWAS were combined through the meta-analysis of four independent studies, one of which is the NIHRBR, with 847 PAH cases and 5048 rare disease controls. The other major contributing study, the US PAH Biobank (PAHB), used a control population with mixed common diseases recruited from outpatient clinics (694 PAH cases and 1560 controls), while the two smaller studies used population-based controls, with 269 and 275 PAH cases and 1068 and 1983 controls in the Pulmonary Hypertension Allele-Associated Risk and British Heart Foundation Pulmonary Arterial Hypertension studies, respectively [4].

### Statistical analyses

To confirm and refine the estimate for the association between RDW and PAH, we combined PAH patients from the Imperial College Pulmonary Hypertension Biobank, the UK PAH Cohort and the VUMC study and compared them to common disease controls from the VUMC recruited in outpatient clinics (supplementary methods and supplementary table S1).

To test for causality between RDW and PAH, we applied a two-sample MR strategy that requires effect estimates for the genetic instrument on the risk factor (here RDW) and the outcome (here PAH) from two non-overlapping datasets (supplementary methods and supplementary figure S2). The genetic instrument for RDW comprised genetic variants associated with RDW levels in the RDW GWAS at a study-specific genome-wide level of significance (p<8.31×10^−9^). In the PAH GWAS, 179 variants (inclusive of 12 proxy variants with a minimum r^2^ of 0.8) out of the 212 independent (r^2^<0.01) RDW quantitative trait loci (QTL) were available after excluding 13 palindromic variants (A/T or C/G) with intermediate allele frequencies (minor allele frequency >45%) to ensure that the effects of the variants for the two traits can be harmonised to the same allele. To obtain the causal estimate, we applied the inverse variance weighted (IVW) [29] and weighted median [30] methods. We assessed heterogeneity between the causal estimates from each QTL using Cochran's Q-test (supplementary methods).

In the primary MR analysis, we included all available genome-wide significant RDW QTL (n=179). The secondary analysis explicitly tested the role of iron deficiency in the RDW–PAH association using five RDW QTL mapped to genes involved in iron homeostasis (*HFE*, *TMPRSS6*, *TFRC* and *TFR2*) from the full set of genome-wide significant RDW QTL (supplementary figure S2). All of these five RDW QTL concomitantly increase serum iron, ferritin and transferrin saturation and decrease transferrin, reflecting systemic iron status (supplementary table S2) and were first reported by an independent GWAS (the Genetics of Iron Status) as genome-wide significant signals for at least two of the aforementioned iron status biomarkers [31]. These five RDW QTL are among the signals which explain the highest proportion of variance in the RDW GWAS, highlighting the importance of iron availability in RDW levels.

Furthermore, we validated the RDW genetic instrument as a proxy for RDW levels in our common disease controls from VUMC. To do so, we regressed the first available RDW measurement on the RDW genetic risk score (GRS) constructed for each individual (supplementary methods) and obtained the proportion of variance explained (R^2^). The R^2^ for the same RDW GRS in the RDW GWAS study populations (UK Biobank (UKB) and INTERVAL) was calculated from the summary statistics of the RDW–QTL associations (supplementary methods).

Since genetic variants typically explain a small proportion of the variability in the associated trait, MR studies often require large sample sizes to detect a non-zero causal effect. Our power to detect a causal association in the current MR analyses was calculated [32] using the R^2^ values estimated in VUMC and those estimated in the RDW GWAS populations (supplementary methods).

## Results

### Defining the association of RDW and PAH

**FIGURE 1:**
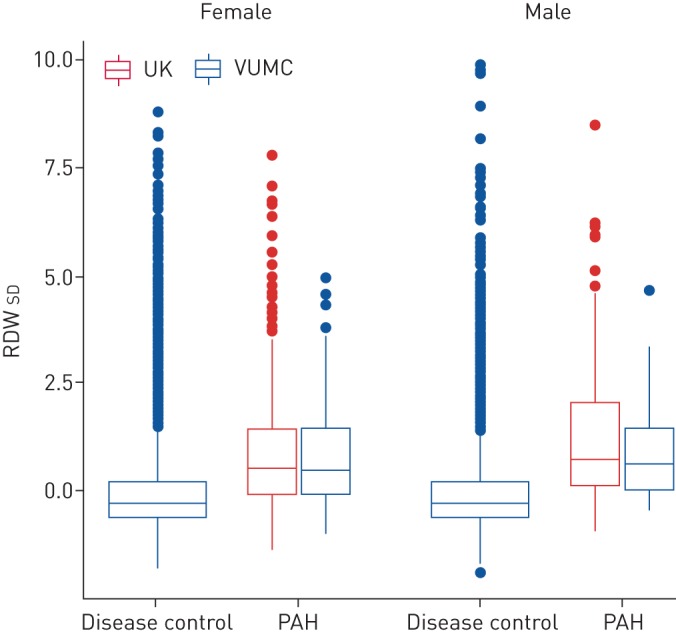
Boxplot of red cell distribution width (RDW) levels in the merged cohort of pulmonary arterial hypertension (PAH) cases (n=642) and the disease control cohort (n=15 889). The bottom and the top lines of the box indicate the 25th and 75th percentiles, while the centre line indicates the median value. The whiskers extend to 1.5 times the interquartile range from both ends of the box with individual points being more extreme observations. VUMC: Vanderbilt University Medical Center.

Within this observational analysis, each standard unit (1.4%) increase in RDW was associated with 90% higher odds of prevalent PAH after adjusting for the effects of age and sex (OR 1.90, 95% CI 1.80–2.01). There were no marked between-cohort differences in RDW levels (figure 1 and supplementary table S1).

### Genetic risk score using RDW QTL

We estimated that the 179 RDW QTL would explain >12% of the variability (R^2^ 12.7%, 95% CI 12.32%–12.99%) in RDW levels in the RDW GWAS population (UK Biobank and INTERVAL) in which they were discovered. The RDW GRS constructed for the VUMC controls explained 2.6% (95% CI 2.17–3.19%) of the variability in the first available RDW measurement (supplementary table S3). The set of five variants specific to iron status explain an estimated 1.7% (95% CI 1.62–1.87%) of the RDW variability in the UKB and INTERVAL populations while they explain 0.7% (95% CI 0.43–0.92%) of the total variability in RDW in the VUMC controls (supplementary table S3). The RDW GWAS study populations had a lower mean RDW level than our common disease controls (UKB and INTERVAL 13.45; VUMC 13.60) and lower standard deviation (UKB and INTERVAL 0.82; VUMC 1.40), which could explain in part differences between the R^2^ estimates in these studies.

### Statistical power to detect causal effect

**FIGURE 2:**
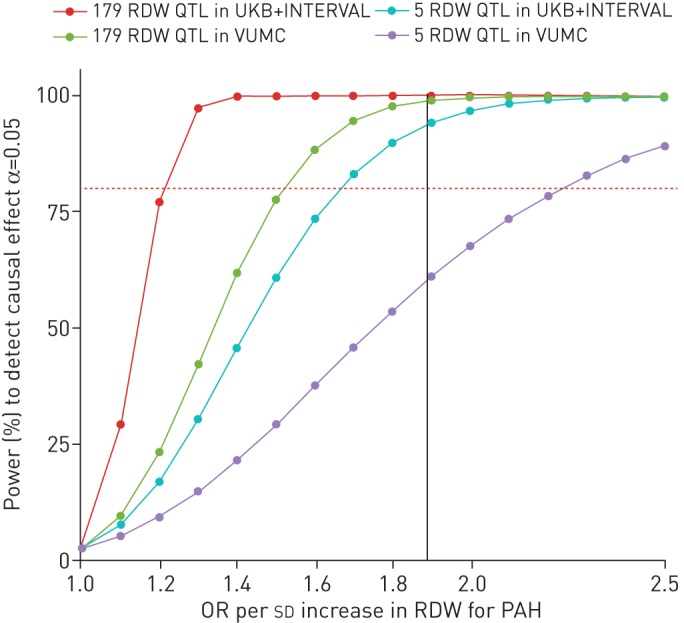
Power (%) to detect a causal association (y-axis) given the size of the true underlying causal effect of one standard unit increase in red cell distribution width (RDW) on pulmonary arterial hypertension (PAH) risk (x-axis). n=11 744 (2085 cases). The effect estimate obtained from the observational study is indicated with the vertical black line at OR 1.90 while the red dotted line marks the desired power of 80%. Red curve: Mendelian randomisation (MR) using all overlapping genome-wide significant variants from the RDW genome-wide association study (GWAS), given the true R^2^ 12% as per estimated in the UK Biobank (UKB) and INTERVAL cohorts; green curve: MR using all genome-wide significant quantitative trait loci (QTL) from the RDW GWAS, given the true R^2^ 2.6% as per estimated in our Vanderbilt University Medical Center (VUMC) control cohort; blue curve: MR using five genome-wide significant variants from the RDW GWAS reflecting systemic iron status, given the true R^2^ 1.7% as per estimated in the UKB and INTERVAL cohorts; purple curve: MR using five genome-wide significant QTL from the RDW GWAS reflecting systemic iron status, given the true R^2^ 0.7% as per estimated in our VUMC control cohort.

With a genetic instrument that explains a relatively high proportion (R^2^ 12%) of the total variation in RDW levels (figure 2, red curve) we have 80% power to detect a causal effect as small as 1.25 (odds ratio). If the true variance explained lies closer to that estimated in the VUMC controls (R^2^ 2.6%; figure 2, green curve), this changes to 1.52. When the variance explained by the genetic instrument is small (R^2^ 1.7%; figure 2, blue curve), we are limited, with our sample size, to an odds ratio of ≥1.7. However, if the effect of RDW calculated in our observational analysis (OR 1.80–2.01) was causal in nature, either of the two MR analyses, based on R^2^ estimates from the RDW GWAS, would have >80% power to detect an effect of that magnitude. Using the estimates based on the VUMC data, the analysis using all 179 RDW QTL (figure 2, green curve) would have sufficient (>80%) power in our sample, while the iron-specific model (figure 2, purple curve) would be underpowered.

### RDW–PAH causal relationship

**FIGURE 3:**
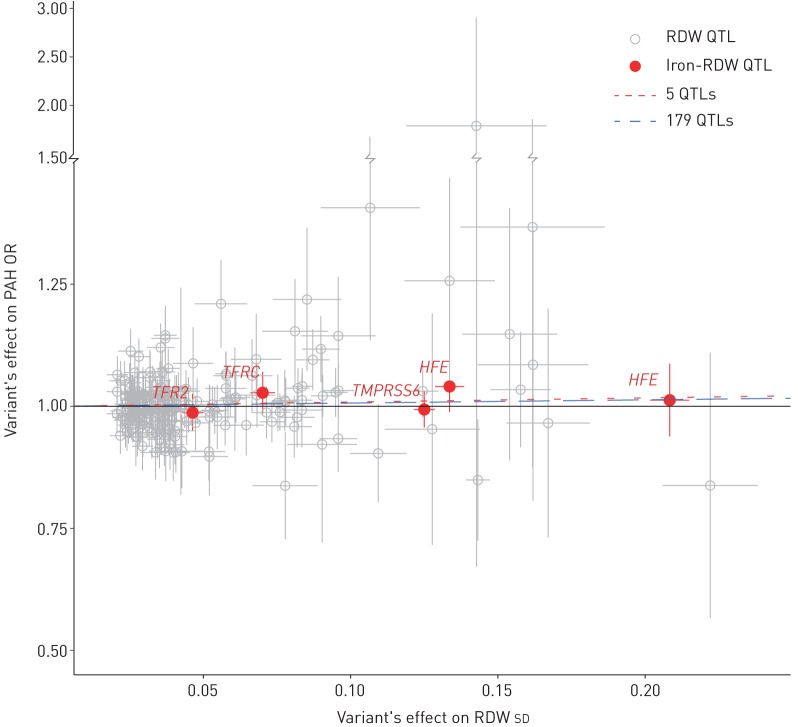
Scatterplot of variant (red cell distribution width (RDW)) associations (x-axis) plotted against variant (pulmonary arterial hypertension (PAH)) associations (y-axis) where each dot represents a single RDW quantitative trait locus (QTL). The effect estimates and their standard errors (grey bars) are given in standard units for RDW and in odds ratios for PAH. The solid black line denotes an odds ratio of 1 (no effect), while the dashed blue line is the overall causal effect from the inverse variance weighted regression using all 179 RDW QTL. The five iron-specific RDW QTL used as instruments in the secondary Mendelian randomisation analysis are labelled with their corresponding gene names and the red dotted line denotes the corresponding causal effect.

We tested for a causal effect of RDW on development of PAH in our primary MR analysis using 179 RDW QTL and found no significant relationship (IVW OR_causal_ 1.07, 95% CI 0.93–1.23; Q p-value 0.57; figure 3). A secondary MR analysis based on five RDW QTL provided no evidence for a causal effect of iron status on PAH (IVW OR_causal_ 1.09, 95% CI 0.77–1.54; Q p-value 0.91; figure 3). The weighted median method, which is more robust to violations of MR instrument assumptions, yielded similar estimates for both the primary (OR_causal_ 1.11, 95% CI 0.89–1.38) and the secondary (OR_causal_ 1.04, 95% CI 0.68–1.59) MR analyses.

If the odds ratio estimated in the primary MR analysis was indicative of the magnitude of a real causal effect, the number of PAH cases needed to detect such a causal effect with a genetic instrument that explains 10% of the variance in RDW would be ∼20 600 (with the same 1:4.6 ratio of cases:controls as in this study) to achieve 80% power at a false-positive rate of 5% (p=0.05). We tested for heterogeneity between causal effects estimated in the four studies contributing to the PAH GWAS separately (supplementary figures S1 and S2) to assess if differences in the nature of their control populations yielded heterogenous effect estimates for the instrumental variants. The two heterogeneity tests on the IVW estimates (main MR: Q=0.83, df=3, p=0.84; secondary MR: Q=0.55, df=3, p=0.91) did not detect considerable variability between the causal effects in the four contributing studies based on a random-effects model (supplementary figures S3 and S4).

## Discussion

We applied a two-sample MR approach to test whether the epidemiological relationship between elevated RDW levels, which are associated with iron deficiency, and PAH is causal in nature. We estimated the effect of RDW on PAH in a large sample of cases and common disease controls. By using genetic variants as instruments for RDW, we found no evidence for a causal effect of RDW on PAH of the magnitude suggested by observational studies.

Previous work has shown that iron deficiency is common in PAH and associated with a poor prognosis, reduced exercise capacity and worsening haemodynamics [9, 11, 13]. A physiological link has been described in healthy volunteers where iron infusion attenuated the rise in pulmonary artery pressure induced by acute hypoxia [33, 34] and in rats where chronic iron deficiency results in pulmonary hypertension [35]. This relationship could be driven by the role of iron in destabilising the hypoxia-inducible factor, thereby deficiency can mimic the hypoxic state [36]. Our study confirmed the association of raised RDW with PAH using controls from a hospital population and cases from multiple centres (with an effect size 1.90 for one standard unit RDW, 1.4%), supporting a recent hypothesis-free phenome-wide analysis which indicated, among several disease descriptors, PAH as the most strongly associated with RDW with a similar effect size (OR 2.0, 95% CI 1.75–2.4 per % increase in RDW) [7]. Our MR analysis was adequately powered to detect a causal role for RDW with an effect of this magnitude. The fact that we did not detect a causal effect at this level suggests that at least some of the observed association is secondary to the disease.

The results of this study may appear to be at odds with previous clinical studies of the efficacy of iron supplementation in PAH patients, which have focused on functional capacity rather than disease pathology. An open-label study of 20 patients with idiopathic PAH with iron deficiency reported improved iron status, 6-min walk distance and quality of life (QoL) 2 months after a single infusion of 1000 mg ferric carboxymaltose [14]. Another open-label study in 15 iron-deficient idiopathic PAH patients reported improvement of iron status, QoL and exercise endurance capacity on cardiopulmonary exercise testing after receiving 1000 mg of intravenous iron [10]. Neither of the clinical studies were placebo controlled, although Viethen *et al.* [14] compared their intervention group to a group of matched iron-replete patients who did not receive iron infusion. It remains possible that iron supplementation in PAH could have benefits through mechanisms distinct from those driving the cardiovascular pathology, for example on muscle function [37].

MR studies using data from large consortia support a causal effect of iron status in other diseases. The genetic instruments (two variants in *HFE* and one variant in *TMPRSS6*) used in these studies were also used in our secondary MR analysis. Gill and co-workers found iron to have a protective effect against coronary artery disease (IVW OR_causal_ 0.94 per sd change in serum iron) [38], but increased the risk of cardioembolic stroke (IVW OR_causal_ 1.16 per sd iron) [39]. The authors suggest the opposing effects of iron status on coronary artery disease and stroke might be due to different underlying mechanisms. Pichler *et al*. [19] have reported that iron protects against the risk of developing Parkinson's disease (IVW OR_causal_ 0.88 per sd iron). Iron deficiency is a common risk factor and these causal effect estimates in common diseases are modest; this study was not powered to detect an effect if this is also true of PAH.

In the light of this MR analysis, alternative explanations for the association of RDW and PAH have to be considered; namely, that PAH causes raised RDW (reverse causation, for example reduced oxygen delivery and/or haemolysis related to PAH may stimulate reticulocytosis, which would increase RDW) or that PAH and elevated RDW are caused by an independent common mechanism. One such mechanism is chronic inflammation, which is a common feature of PAH [40] and leads to intracellular sequestration of iron. Other mechanisms which may modify RDW, such as folate or vitamin B12 could be studied for their association with PAH. To directly test whether PAH is causal for raised RDW levels a stronger genetic instrument for PAH is required than the currently known common variation identified by PAH GWAS.

An important strength of our study lies in the sample size available with phenotype and genetic data achieved through extensive collaboration. Given the rarity of PAH, data had to be pooled from several centres to allow the investigation of common genetic variation in PAH and to test causal relationships. Our study has some limitations. The control population for our observational study was not specifically selected to represent a population at risk of developing PAH. An example of a population at risk of PAH are relatives of patients with pathogenic *BMPR2* mutations (and other rare pathogenic variants). Given the reduced penetrance of familial/heritable PAH (∼42% in females and ∼14% in males carrying known mutations) [41], following-up the families of affected individuals, especially relatives harbouring mutations associated with PAH, would be an invaluable source to identify environmental triggers of PAH.

Despite our efforts to exclude controls with conditions that probably affect RDW and to use the first available RDW measurement, we expect genetic effects of RDW levels to differ between individuals with common diseases and a cohort of healthy volunteers. The R^2^ of a genetic variant describes the variance explained in the phenotype in a given population at a given time. Therefore, there can be no single population parameter that applies to multiple populations or the same population at multiple time points. Although the R^2^ estimated in the RDW GWAS is likely to be upwardly biased (since it contains the discovery as well as the replication samples), it might reflect better the extent to which genetic variation influences long-term RDW levels in disease-free populations than the R^2^ estimate from the VUMC disease controls, who may be expected to have more variable RDW levels due to comorbidities. This highlights a potential challenge in estimating power for MR studies. We now estimate that >20 000 PAH patients would be required to detect any likely causal effect of iron deficiency.

It is important to note that in our MR analyses, causal effects were estimated using the results of the PAH GWAS [4]. Four independent studies contributed to the overall results of the PAH GWAS comparing allele frequencies of their PAH cases to control cohorts selected according to different criteria. The NIHRBR study used a control population with a mixture of rare diseases, while the other major contributing study from the United States used mixed common disease controls. The two smaller contributing studies used population-based controls; a preferable design for estimating the effects of surrogate genetic variants for common risk factors. Selection bias can affect the estimates of the instrumental genetic variants on disease susceptibility. This is especially true if the control cohort is enriched for conditions also affected by the risk factor of interest.

### Conclusions

There is strong observational evidence for an association between elevated RDW, a surrogate for iron deficiency, and PAH. However, this MR analysis does not indicate that RDW is causally linked to disease development. Our study was powered to detect a causal effect similar in size to that observed. A more modest causal effect remains possible, but a significantly larger study population would be required to detect it. Extending international collaborations and careful follow-up of populations at risk will allow increasingly sophisticated study designs to investigate causal relationships, shared underlying mechanisms with other conditions and overall genetic susceptibility in PAH.

## Supplementary material

10.1183/13993003.01486-2019.Supp1 **Please note:** supplementary material is not edited by the Editorial Office, and is uploaded as it has been supplied by the author.
Supplementary material ERJ-01486-2019.SUPPLEMENT

## Shareable PDF

10.1183/13993003.01486-2019.Shareable1 This one-page PDF can be shared freely online.
Shareable PDF ERJ-01486-2019.Shareable
