# Supplementary material for: Mendelian randomisation analysis of red cell distribution width in pulmonary arterial hypertension
Source: Eur Respir J. 2020 Feb 13;55(2):1901486. doi: 10.1183/13993003.01486-2019 (PMC7015630; doi:10.1183/13993003.01486-2019)
Supplement: Supplementary file 1 [file ERJ-01486-2019.SUPPLEMENT.pdf]

Supplementary Appendix
for

Mendelian randomization analysis of red cell distribution width in pulmonary arterial hypertension

Anna Ulrich *et al.*

Table of Contents

Supplementary Methods 2
RDW and PAH association 2
Mendelian randomization; causal effect estimation using MR 3
Inverse-variance weighted method 3
Weighted median estimator 3
Quality control and imputation procedures of VUMC genotype data 5
Genetic risk score (GRS) derivation and calculation of variance explained (R^2) from individual-level data 5
R^2 calculation from GWAS summary-level data 7
Supplementary Table and Figure Legends 8
Supplementary Tables and Figures 10

Supplementary Methods

RDW and PAH association

RDW values were natural log-transformed and for ease of interpretation z-score normalized to have a mean of zero and a standard deviation of one. The association between RDW levels and PAH was tested in a logistic regression framework adjusted for age and sex (Supplementary Table S1). For PAH cases we used closest to diagnosis RDW measurements. For controls we retrieved the first available RDW measurement.

We excluded individuals from non-white ethnic backgrounds to avoid potential bias from ethnicity effects. Since RDW is known to be elevated in a number of diseases, we excluded all individuals with either of the following in their medical history: polycystic kidney disease, chronic kidney disease, liver disease and transfusion therapy received. Furthermore, children and adolescents under the age of 18 as well as individuals with extreme RDW values (below 10%, N=20 or above 30%, N=15) were not included in the analysis. Out of the 35 excluded for extreme RDW, 14 were cases from VUMC in the range of 43-55 and 20 were all below 10 from one of the NIHRBR centers. Both centers confirmed reporting errors for these samples (*Supplementary Figure S1*).

Mendelian randomization; causal effect estimation using MR

Alleles were aligned to correspond to an increase in RDW followed by the harmonization of the effects. The causal effect was estimated with the inverse variance weighted (IVW) and weighted median estimator (WM) methods as implemented in the MR-Base software (1).

Inverse-variance weighted method

We used the conventional inverse variance weighted (IVW) method for estimating the causal effect. The IVW method is efficient when all variants in the genetic instrument are valid instruments. Briefly, each variant in the genetic instrument provided a causal estimate calculated by simply dividing the variant's effect on PAH by the variant's effect on RDW (ratio of coefficients or Wald ratio). These individual causal estimates were then meta-analyzed in a fixed-effects model weighted by the reciprocal of the standard error of the variant association with PAH.

This is equivalent to regressing the variant-RDW estimates on the variant-PAH estimates with the above-mentioned weighting whilst forcing the regression line to pass through the origin.

The causal estimate from the IVW method (β_{IVW}) is:

$$\beta_{IVW}=\frac{\sum_{k=1}^{K} X_k Y_k \sigma_{Yk}^{-2}}{\sum_{k=1}^{K} X_k^2 \sigma_{Yk}^{-2}}$$

Where:
k is an index for each of the variants used in the two-sample MR analysis
X is the effect estimate on the exposure (RDW) as reported in the RDW GWAS summary statistics
Y is the effect estimate on the outcome (PAH) as reported in the PAH GWAS summary statistics

The standard error of the causal estimate is:

$$se(\beta_{IVW}) = \sqrt{\frac{1}{\sum_{k=1}^{K} X_k^2 \sigma_{Yk}^{-2}}}$$

Weighted median estimator

We used the WM estimator to allow for up to (but not including 50%) of the variants in our genetic instrument to be invalid instruments.

Analogously to the IVW method, a Wald ratio (see above) is calculated for each variant. These Wald ratios are then ordered and weighted by the same weights used in the IVW method (see above). Let wj be the weight of the jth ordered Wald ratio estimate.

$$s_j = \sum_{k=1}^{j} w_k$$

Where:

k is an index for each of the variants used in the two-sample MR analysis

w is the weight of the variant

s_j is the sum of weights up to and including the jth Wald ratio estimate

The weights are standardized, so that the sum of weights is 1. The WM estimator is the median of the empirical distribution of weighted Wald ratios. Each Wald ratio is the $100(s_j - \frac{w_j}{2})$th percentile of this distribution.

Quality control and imputation procedures of VUMC genotype data

VUMC participants were genotyped in 6 batches (30,886 in total) using the Infinium Expanded Multi-Ethnic Global Array-8 (MEGA-ex) array (Illumina, San Diego, California, US).

Variant QC (pre-imputation): Variants were excluded if they had a low call rate (< 95%), deviated from the Hardy-Weinberg equilibrium ($P \leq 0.00005$), were rare (minor allele frequency ≤ 1%) or had more than two alleles.

Sample QC: Individuals with high proportions (> 5%) of missing genotype data, unresolved sex discrepancies (discordant phenotype-genotype sex information), heterozygosity outliers, self-reported and/or principal component-based ethnic outliers, intentional duplicates and related individuals (PI-HAT > 0.2) were excluded.

Imputation of non-genotyped variants: The filtered genotype array data was imputed to the Haplotype Reference Consortium panel using the free Sanger Imputation Service provided by the Wellcome Sanger Institute (2).

Imputed variants were further filtered for deviations from the Hardy-Weinberg equilibrium ($P \leq 0.00005$), rare variants (minor allele frequency ≤ 1%) which are often poorly imputed and other low-quality variants with an INFO score lower than 0.9.

Genetic risk score (GRS) derivation and calculation of variance explained (R^2) from individual-level data

Weighted genetic risk scores (GRS) comprising the single nucleotide polymorphisms (SNPs) from the RDW genetic instrument were regressed onto the first RDW values which provided the coefficient of determination (R^2) as an estimate for the correlation between RDW and the RDW GRS in our population. The GRSs can be derived by summing the effect alleles multiplied by the effect size at each of the variants (3).

GRS were calculated using the software PRSice-2 (3). Genotypes for the 179 SNPs in the RDW GRS were extracted from the imputed VUMC controls dataset. The same inclusion criteria as for our observational study were applied. Out of the 15,889 VUMC controls included in the observational study, 14,964 had genetic data that passed standard variant and sample quality control.

The GRS for an individual is the summation of the effect (trait-increasing) alleles (0, 1 or 2) weighted by the effect size of the variant taken from the genome-wide significant summary statistics of the RDW GWAS (4). We used an additive model meaning that homozygotes for the effect allele had twice the increase in RDW levels as the heterozygotes. This was in line with the model used in the RDW GWAS.

The following models were used to assess the validity of the RDW GRS as a proxy for RDW levels:

Full model: *RDW ~ GRS + sex + age + principal components (1st and 2nd) + batch (study specific)*

Null model: *sex + age + principal components (1st and 2nd) + batch (study specific)*

The R^2 for the GRS alone is calculated by subtracting the R^2 of the model not containing the GRS (null model) from the R^2 of the full model.

The confidence intervals for the GRS R^2 were computed using the adjusted bootstrap percentile method as implemented in the R software package ‘boot’ (5) (number of replicates = 20,000).

R^2 calculation from GWAS summary-level data

R^2 was calculated for each independent variant based from the publicly available summary statistics of the RDW GWAS in the discovery and replication populations (4). These individual estimates were then summed to give the overall variance explained by the RDW instrument.

$$R^2 = \sum_{k=1}^{K} \frac{Nsample_k + 1}{Nsample_k} \times \frac{Z_k^2}{Z_k^2 + Nsample_k} - \frac{1}{Nsample_k}$$

Where:
k is an index for each of the variants used in the two-sample MR analysis
Z is the Z-statistic as reported in the RDW GWAS summary statistics
$Nsample$ is the sample size as reported in the RDW GWAS summary statistics

The standard error for the R2 estimate was calculated as shown below:

$$SE_{R^2} = \sum_{k=1}^{K} \sqrt{\left(\frac{2}{Nsample_k}\right) \times \left(2 \times R_k^2 + \frac{1}{Nsample_k}\right)}$$

Supplementary Table and Figure Legends

Supplementary Table S1 Characteristics of the study population used for estimating/assessing the association between RDW and PAH, stratified by sex. PAH – Pulmonary Arterial Hypertension, VUMC - Vanderbilt Institute for Clinical and Translational Research, NIHRBR - UK National Institute for Health Research BioResource, RDW – red cell distribution width.

Supplementary Table S2 Logistic regression model predicting PAH disease status. We report the results of the adjusted model in the paper.

Supplementary Table S3 Five variants selected from the RDW GWAS based on their effects on systemic iron status. This table presents the effect estimates of these variants on RDW as reported by Astle et al. on RDW (Effect estimate per RDW SD; Effect estimate p-value). *The effects of these variants for the same allele go in the opposite direction on serum iron as reported by the Genetics of Iron Status GWAS (6). Elevated RDW can reflect iron deficiency which presents with decreased serum iron levels. In the Genetics of Iron Status GWAS, the two HFE variants reached genome-wide significance ($P< 5\times10^{-8}$) for all four (serum iron, transferrin, transferrin saturation, ferritin) iron status biomarkers, TMPRSS6 reached it for all but transferrin, TFRC reached it for transferrin and transferrin saturation, while TFR2 reached it for iron and transferrin saturation.

Supplementary Table S4 Summary of the RDW GRS models. The null model corresponds to the linear regression model specified above without the GRS. The R2 of the null models are identical since the sample and the covariates are the same. P.value is the significance value of the model fit (F-test). Empirical p-values that account for multiple testing and overfitting were obtained through permutation tests (n=20,000).

Supplementary Figure S1 Flow diagram of the exclusion steps in the UK centers and the Vanderbilt University Medical Centre (VUMC) of the two cohorts (UK PAH Cohort and VUMC) participating in the RDW and PAH association analysis as described in the Supplementary methods.

Supplementary Figure S2 Mendelian Randomization (MR) analyses of red cell distribution width (RDW) and pulmonary arterial hypertension (PAH). A two-sample design was used where effect estimates for the instrumental genetic variants were taken from two non-overlapping populations. RDW QTL and their effect estimates were taken from the largest-to-date population based RDW genetic association study (GWAS) (4). Effect estimates for the RDW QTL on PAH susceptibility were obtained from the largest-to-date PAH GWAS (6). The primary MR analysis included all 179 RDW QTL while the secondary MR analysis was restricted to five out of the 179 RDW QTL acting via iron status (Supplementary Table S3).

Supplementary Figure S3 Individual MR causal estimates (IVW) for the main MR analysis of association between RDW and development of PAH - using all available RDW SNPs - from the four contributing studies in PAH GWAS. PAH ORs per one standard unit increase in RDW (dot) with the corresponding lower and upper 95% confidence intervals (horizontal line). The result of the BHFPAH (IVW causal OR = 1.54, 95% CI = 1.06 – 2.23) did not survive the correction for multiple testing and was driven by the causal estimate of one variant (rs6883412). PHAAR: Pulmonary Hypertension Allele-Associated Risk (269 PAH cases, 1068 controls). PAHB: US National Biological Sample and Data Repository for Pulmonary Arterial Hypertension (694 PAH cases, 1560 controls). NIHRBR: UK National Institute for Health Research BioResource (847 PAH cases, 5048 controls). BHFPAH: British Heart Foundation Pulmonary Arterial Hypertension (275 PAH cases, 1983 controls). Meta-analyzed: overall results of PAH GWAS including all four studies.

Supplementary Figure S4 Individual MR causal estimates (IVW) for the secondary MR analysis of association of RDW to development of PAH – using 5 SNPs related to systemic iron status - from the four contributing studies in PAH GWAS. PAH ORs per one standard unit increase in RDW (dot) with the corresponding lower and upper 95% Cis (horizontal line). PHAAR: Pulmonary Hypertension Allele-Associated Risk (269 PAH cases, 1068 controls). PAHB: US National Biological ample and Data Repository for Pulmonary Arterial Hypertension (694 PAH cases, 1560 controls). NIHRBR: UK National Institute for Health Research BioResource (847 PAH cases, 5048 controls). BHFPAH: British Heart Foundation Pulmonary Arterial Hypertension (275 PAH cases, 1983 controls). Meta-analyzed: overall results of PAH GWAS including all four studies.

Supplementary Tables and Figures

	PAH (118 VUMC; 524 NIHRBR)		Controls (15,889 VUMC)	
	Female (%)	Male (%)	Female (%)	Male (%)
N	445 (69)	197 (31)	8,539 (54)	7,350 (46)
RDW (mean/SD)	15.1/2.20	15.5/2.33	13.6/1.41	13.6/1.39
Age (mean/SD)	52.4/17.6	58.2/16.2	54.3/16.1	58.1/14.7

Supplementary Table S2~~1~~ Characteristics of the study population used for estimating/assessing the association between RDW and PAH, stratified by sex. PAH – Pulmonary Arterial Hypertension, VUMC - Vanderbilt University Medical Centre, NIHRBR - UK National Institute for Health Research BioResource, RDW – red cell distribution width.

Variable	Unadjusted		Adjusted	
	OR	95% CI	OR	95% CI
RDW (SD)	1.85	1.75 – 1.94	1.90	1.80 – 2.01
Age	-	-	0.98	0.98 – 0.99
Sex *(base=female)*	-	-	0.54	0.45 – 0.64

Supplementary Table S2 Logistic regression model predicting PAH disease status. We report the results of the adjusted model in the paper.

Variant information				RDW GWAS<br>Astle et al.		Genetics of Iron Status GWAS<br>Benyamin et al.								
Gene	Lead variant ID	Effect Allele	Allele Frequency in UKB and INTERVAL	beta RDW	*P* - value	Proxy variant ID*	beta iron	*P* – value iron	beta ferritin	*P* – value ferritin	beta TSAT	*P* – value TSAT	beta TF	P – value TF
HFE	rs144861591	C	0.92	0.21	6.50×10^{-216}	rs1800562	-0.37	4.0×10^{-77}	-0.21	1.4×10^{-29}	-0.58	1.5×10^{-178}	0.55	1.3×10^{-153}
TMPRSS6	rs855791	A	0.44	0.13	9.79×10^{-271}	-	-0.19	4.3×10^{-77}	-0.05	5.8×10^{-8}	-0.19	3.5×10^{-80}	0.04	1.3×10^{-4}
HFE	rs198851	G	0.85	0.13	2.55×10^{-161}	-	-0.19	1.6×10^{-40}	-0.06	3.6×10^{-6}	-0.23	4.7×10^{-59}	0.12	3.0×10^{-17}
TFRC	rs7619708	C	0.24	0.07	4.35×10^{-64}	rs6583288	0.03	1.2×10^{-2}	0.004	7.3×10^{-1}	0.05	3.8×10^{-6}	-0.06	$3.8 \text{ X } 10^{-8}$
TFR2	rs9801017	G	0.37	0.05	9.40×10^{-37}	rs7385804	-0.06	7.2×10^{-8}	-0.02	2.5×10^{-2}	-0.05	1.8×10^{-7}	0.01	$4.0 \text{ X } 10^{-1}$

*Supplementary Table S3 Five variants selected from the RDW GWAS based on their effects on systemic iron status. This table presents the effect estimates of these variants on RDW as reported by Astle et al. on RDW (Effect estimate per RDW SD; Effect estimate p-value). Elevated RDW can reflect iron deficiency which presents with decreased serum iron levels. In the Genetics of Iron Status GWAS, the two HFE variants reached genome-wide significance ($P< 5\times10^{-8}$) for all four (serum iron, transferrin, transferrin saturation, ferritin) iron status biomarkers, TMPRSS6 reached it for all but transferrin, TFRC reached it for transferrin and transferrin saturation, while TFR2 reached it for iron and transferrin saturation. The betas for RDW and the iron biomarkers from Benyamin et al. are reported in standard units. RDW = red cell distribution width; TSAT = transferrin saturation; TF = transferrin. *Where the lead RDW variant for the locus was not available in the Genetics of Iron Status GWAS we listed the results of a suitable proxy variant in strong linkage disequilibrium ($r^2 \geq 0.8$) with the RDW lead variant.*

RDW GRS	GRS R^2	Null R^2	P value	Empirical P value
179 RDW QTLs	0.0264	0.054	2.4×10^{-94}	5.0×10^{-5}
5 RDW QTLs	0.0065	0.054	3.3×10^{-24}	5.0×10^{-5}

Supplementary Table S4 Summary of the RDW GRS models. The null model corresponds to the linear regression model without the GRS. The R^2 of the null models are identical since the sample and the covariates are the same. P value is the significance value of the model fit (F-test). Empirical p-values that account for multiple testing and overfitting were obtained through permutation tests (n=20,000).

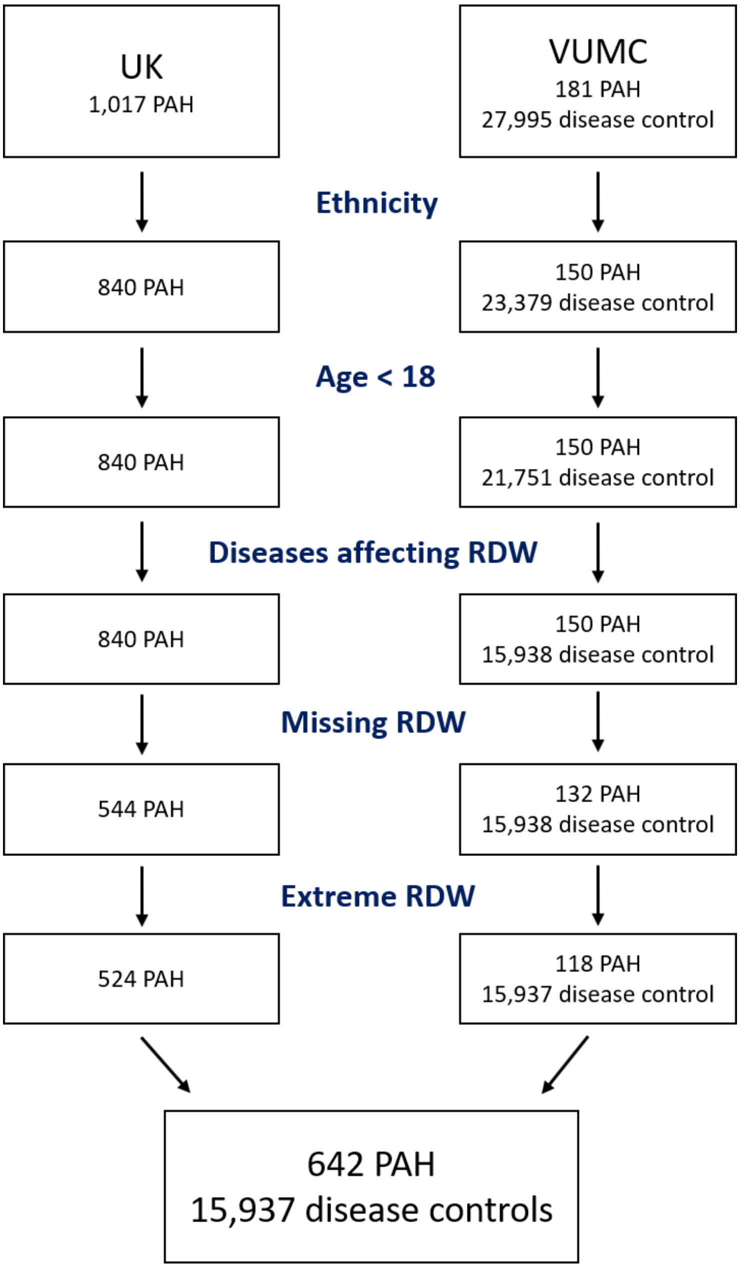

Supplementary Figure S1 Flow diagram of the exclusion steps in the UK centers and the Vanderbilt University Medical Centre (VUMC) participating in the RDW and PAH association analysis as described in the Supplementary methods.

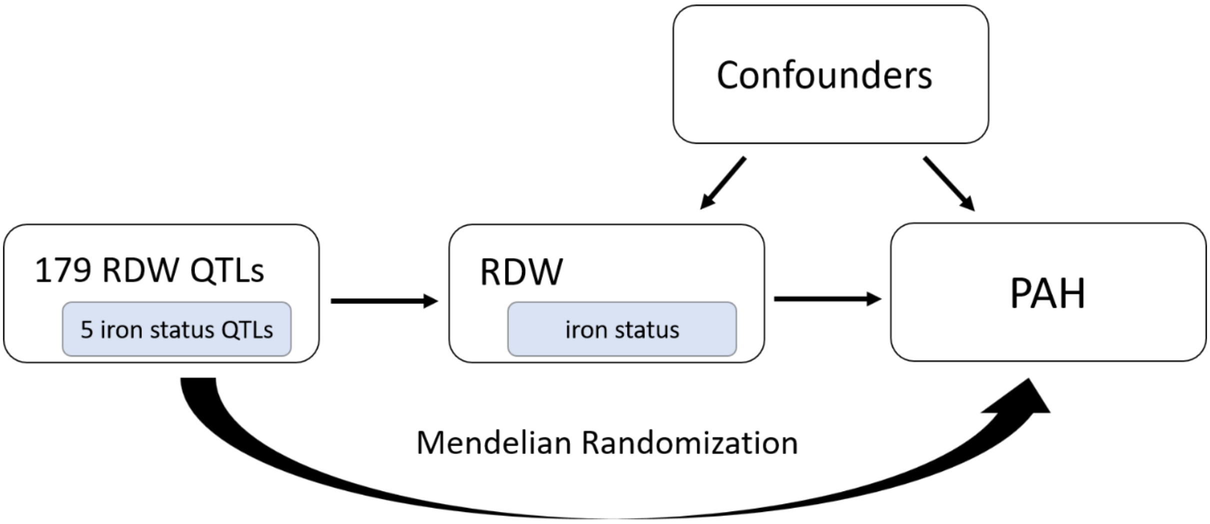

Supplementary Figure S2 Mendelian Randomization (MR) analyses of red cell distribution width (RDW) and pulmonary arterial hypertension (PAH). A two-sample design was used where effect estimates for the instrumental genetic variants were taken from two non-overlapping populations. RDW QTL and their effect estimates were taken from the largest-to-date population based RDW genetic association study (GWAS) (4). Effect estimates for the RDW QTL on PAH susceptibility were obtained from the largest-to-date PAH GWAS (7). The primary MR analysis included all 179 RDW QTL while the secondary MR analysis was restricted to five out of the 179 RDW QTL acting via iron status (Supplementary Table S3).

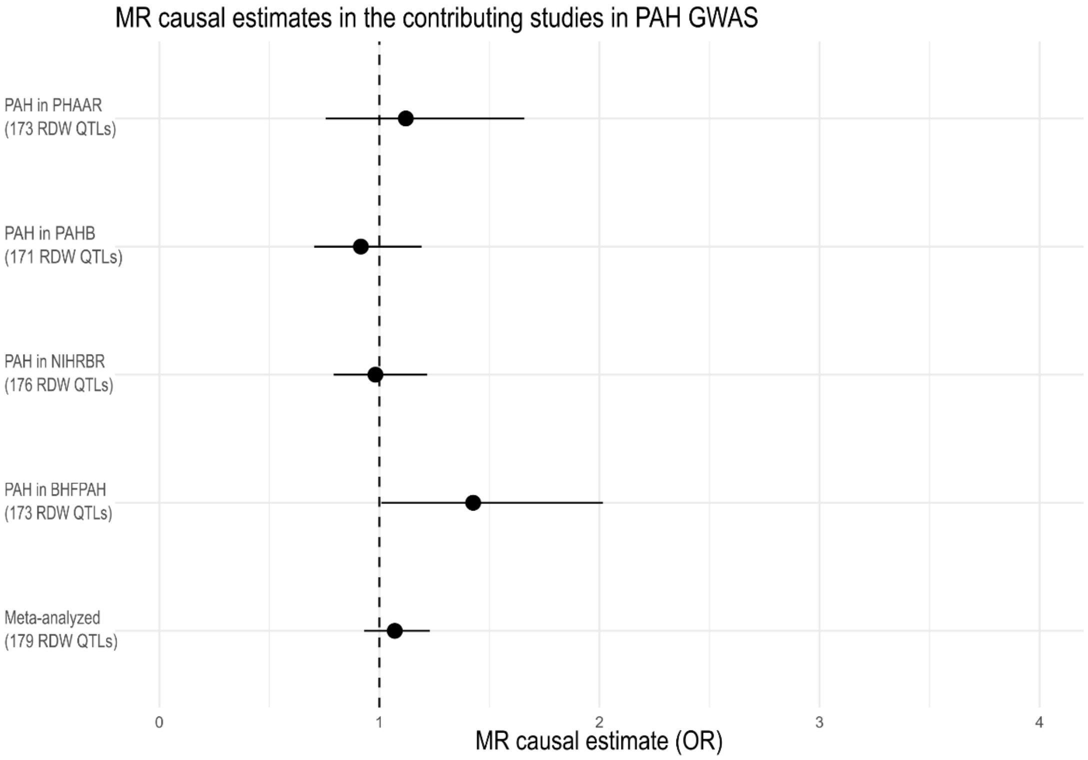

Supplementary Figure S3 Individual inverse variance weighted MR causal estimates for the main MR analysis of association between RDW and development of PAH - using all available RDW QTLs including suitable proxy variants with a minimum r^2 of 0.8 in each study - from the four contributing

studies in PAH GWAS. PAH ORs per one standard unit increase in RDW (dot) with the corresponding lower and upper 95% confidence intervals (horizontal line). The result of the BHFPAH (OR causal = 1.43, 95% CI = 1.01 – 2.02) did not survive the correction for multiple testing and was driven by the causal estimate of one variant (rs6883412). PHAAR: Pulmonary Hypertension Allele-Associated Risk (269 PAH cases, 1068 controls). PAHB: US National Biological Sample and Data Repository for Pulmonary Arterial Hypertension (694 PAH cases, 1560 controls). NIHRBR: UK National Institute for Health Research BioResource (847 PAH cases, 5048 controls). BHFPAH: British Heart Foundation Pulmonary Arterial Hypertension (275 PAH cases, 1983 controls). Meta-analyzed: overall results of PAH GWAS including all four studies.

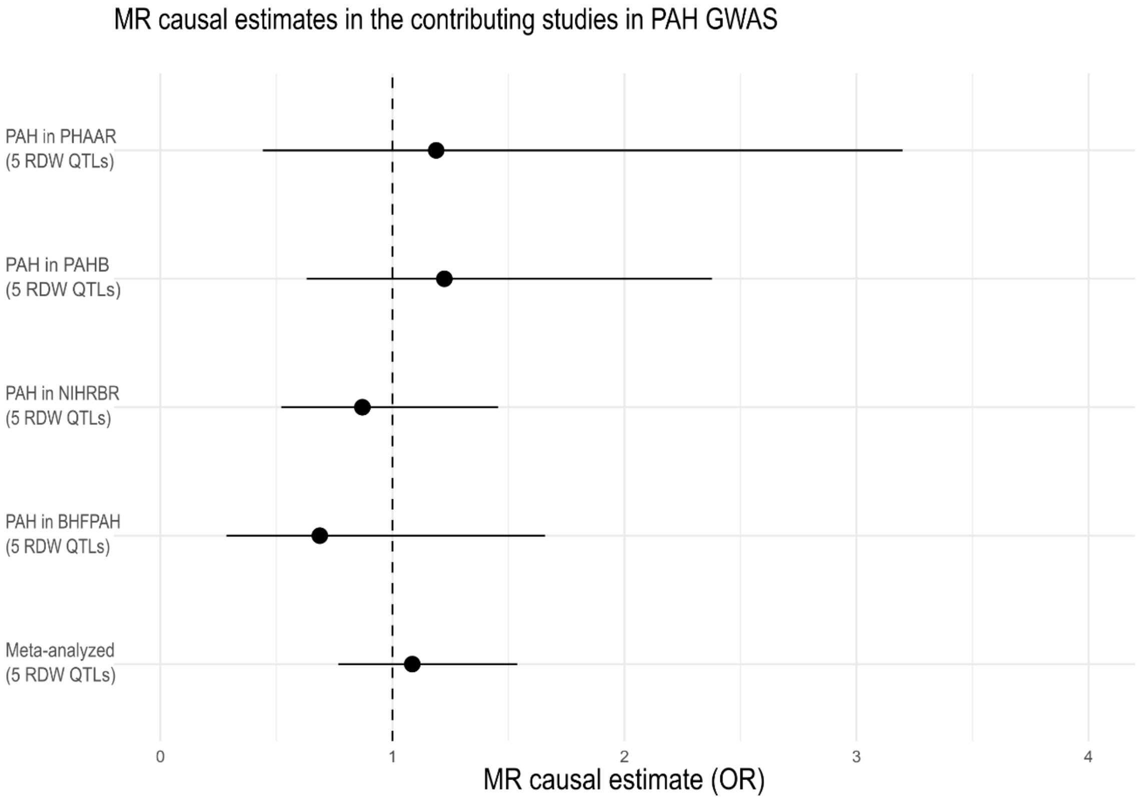

Supplementary Figure S4 Individual MR causal estimates (IVW) for the secondary MR analysis of association of RDW to development of PAH – using 5 RDW QTLs related to systemic iron status - from the four contributing studies in PAH GWAS. PAH ORs per one standard unit increase in RDW (dot) with the corresponding lower and upper 95% confidence intervals (horizontal line). PHAAR: Pulmonary Hypertension Allele-Associated Risk (269 PAH cases, 1068 controls). PAHB: US National Biological Sample and Data Repository for Pulmonary Arterial Hypertension (694 PAH cases, 1560 controls). NIHRBR: UK National Institute for Health Research BioResource (847 PAH cases, 5048 controls). BHFPAH: British Heart Foundation Pulmonary Arterial Hypertension (275 PAH cases, 1983 controls). Meta-analyzed: overall results of PAH GWAS including all four studies.

References

1. Hemani G, Zheng J, Elsworth B, Wade KH, Haberland V, Baird D, et al. The MR-Base platform supports systematic causal inference across the human phenome. eLife. 2018;7:e34408.
2. the Haplotype Reference C, McCarthy S, Das S, Kretzschmar W, Delaneau O, Wood AR, et al. A reference panel of 64,976 haplotypes for genotype imputation. Nature Genetics. 2016;48:1279.
3. Euesden J, Lewis CM, O'Reilly PF. PRSice: Polygenic Risk Score software. Bioinformatics. 2015;31(9):1466-8.
4. Astle WJ, Elding H, Jiang T, Allen D, Ruklisa D, Mann AL, et al. The Allelic Landscape of Human Blood Cell Trait Variation and Links to Common Complex Disease. Cell. 2016;167(5):1415-29 e19.
5. Canty A, Ripley B. boot: Bootstrap R (S-Plus) Functions. R package version 1.3-23. ed2019.
6. Benyamin B, Esko T, Ried JS, Radhakrishnan A, Vermeulen SH, Traglia M, et al. Novel loci affecting iron homeostasis and their effects in individuals at risk for hemochromatosis. Nat Commun. 2014;5:4926.
7. Rhodes CJ, Batai K, Bleda M, Haimel M, Southgate L, Germain M, et al. Genetic determinants of risk in pulmonary arterial hypertension: international genome-wide association studies and meta-analysis. The Lancet Respiratory Medicine. 2018.

NIHR BioResource Collaborators

NIHR BioResource – Rare Disease Consortium[1], Julian Adlard[2], Munaza Ahmed[3], Tim Aitman[4,5], Hana Alachkar[6], David Allsup[7], Jeff Almeida-King[8], Philip Ancliff[9], Richard Antrobus[10], Ruth Armstrong[11,12,13], Gavin Arno[14,15], Sofie Ashford[1,16], William Astle[1,16,17], Anthony Attwood[1,16], Chris Babbs[18,19], Tamam Bakchoul[20], Tadbir Bariana[21,22], Julian Barwell[23,24], David Bennett[25], David Bentley[26], Agnieszka Bierzynska[27], Tina Biss[28], Marta Bleda[29], Harm Bogaard[30], Christian Bourne[26], Sara Boyce[31], John Bradley[1], Gerome Breen[32,33], Paul Brennan[34,35], Carole Brewer[36], Matthew Brown[1,16], Michael Browning[37], Rachel Buchan[38,39], Matthew Buckland[40], Teofila Bueser[41,42,43], Siobhan Burns[40], Oliver Burren[29], Paul Calleja[44], Gerald Carr-White[42], Keren Carss[1,16], Ruth Casey[11,12,13], Mark Caulfield[45], John Chambers[46,47], Jennifer Chambers[48,49], Floria Cheng[49], Patrick F Chinnery[1,50,51], Martin Christian[52], Colin Church[53], Naomi Clements Brod[1,16], Gerry Coghlan[40], Elizabeth Colby[27], Trevor Cole[54], Janine Collins[55], Peter Collins[56], Camilla Colombo[26], Robin Condliffe[57], Stuart Cook[38,58,59,60], Terry Cook[61], Nichola Cooper[62], Paul Corris[63,64], Abigail Crisp-Hihn[1,16], Nicola Curry[65], Cesare Danesino[66], Matthew Daniels[67,68], Louise Daugherty[1,16], John Davis[1,16], Sri V V Deevi[1,16], Timothy Dent[68], Eleanor Dewhurst[1,16], Peter Dixon[48], Kate Downes[1,16], Anna Drazyk[69], Elizabeth Drewe[70], Tina Dutt[71], David Edgar[72], Karen Edwards[1,16], William Egner[73], Wendy Erber[74], Marie Erwood[1,16], Maria C Estiu[75], Gillian Evans[76], Dafydd Gareth Evans[77], Tamara Everington[78], Mélanie Eyries[79], Remi Favier[80,81,82], Debra Fletcher[1,16], James Fox[1,16], Amy Frary[1,16], Courtney French[83], Kathleen Freson[84], Mattia Frontini[1,16], Daniel Gale[85], Henning Gall[86], Claire Geoghegan[26], Terry Gerighty[26], Stefano Ghio[87], Hossein-Ardeschir Ghofrani[62,86], Simon Gibbs[38], Kimberley Gilmour[88], Barbara Girerd[89,90,91], Sarah Goddard[92], Keith Gomez[21,22], Pavels Gordins[93], David Gosal[6], Stefan Gräf[1,16,29], Luigi Grassi[1,16], Daniel Greene[1,16,17], Lynn Greenhalgh[94], Andreas Greinacher[95], Paolo Gresele[96], Philip Griffiths[97,98], Sofia Grigoriadou[99], Russell Grocock[26], Detelina Grozeva[11], Scott Hackett[100], Charaka Hadinnapola[29], William Hague[101], Matthias Haimel[1,16,29], Matthew Hall[70], Helen Hanson[94], Kirsty Harkness[102], Andrew Harper[38,67,103], Claire Harris[64], Daniel Hart[55], Ahamad Hassan[104], Grant Hayman[105], Alex Henderson[106], Jonathan Hoffmann[54], Rita Horvath[107,108], Arjan Houweling[30], Luke Howard[38], Fengyuan Hu[1,16], Gavin Hudson[107], Joseph Hughes[26], Aarnoud Huissoon[100], Marc Humbert[89,90,91], Sean Humphray[26], Sarah Hunter[26], Matthew Hurles[109], Louise Izatt[110], Roger James[1,16], Sally Johnson[111], Stephen Jolles[112,113], Jennifer Jolley[1,16], Neringa Jurkute[14,22], Mary Kasanicki[114], Hanadi Kazkaz[115], Rashid Kazmi[31], Peter Kelleher[39], David Kiely[57], Nathalie Kingston[1], Robert Klima[44], Myrto Kostadima[1,16], Gabor Kovacs[116,117], Ania Koziell[118,119], Roman Kreuzhuber[1,16], Taco Kuijpers[120,121], Ajith Kumar[3], Dinakantha Kumararatne[114], Manju Kurian[122,123], Michael Laffan[124,125], Fiona Lalloo[77], Michele Lambert[126,127], Hana Lango Allen[1,16], Allan Lawrie[128], Mark Layton[124], Claire Lentaigne[124,125], Adam Levine[85], Rachel Linger[1,16], Hilary Longhurst[99], Eleni Louka[18,19], Robert MacKenzie Ross[129], Bella Madan[130], Eamonn Maher[11,131], Jesmeen Maimaris[88], Sarah Mangles[132], Rutendo Mapeta[1,16], Kevin Marchbank[64], Stephen Marks[9], Hugh S Markus[69], Hanns-Ulrich Marschall[133], Andrew Marshall[134,135,136], Jennifer Martin[1,16,29], Mary Mathias[137], Emma Matthews[22,138], Heather Maxwell[139], Paul McAlinden[64], Mark McCarthy[19,103,140], Stuart Meacham[1,16], Adam Mead[141], Karyn Megy[1,16], Sarju Mehta[142], Michel Michaelides[14], Carolyn Millar[124,125], Shahin Moledina[9], David Montani[89,90,91], Tony Moore[14,15], Nicholas Morrell[1,29], Monika Mozere[85], MPGN/C3 Glomerulopathy Rare Renal Disease group[143], Keith Muir[144], Andrew Mumford[145,146], Michael Newnham[29], Jennifer O'Sullivan[130], Samya Obaji[56], Steven Okoli[18,19], Andrea Olschewski[116], Horst Olschewski[116,117], Kai Ren Ong[54], Elizabeth Ormondroyd[67,68], Willem Ouwehand[1,16], Sofia Papadia[1,16], Soo-Mi Park[12,13,147], David Parry[5], Joan Paterson[11,12,13], Andrew Peacock[53], John Peden[26], Kathelijne Peerlinck[84], Christopher Penkett[1,16], Joanna Pepke-Zaba[148],

Romina Petersen[1,16], Angela Pyle[107], Stuart Rankin[44], Anupama Rao[9], F Lucy Raymond[1,11], Paula Rayner-Matthews[1,16], Christine Rees[26], Augusto Rendon[45], Tara Renton[43], Andrew Rice[149,150], Sylvia Richardson[17], Alex Richter[10], Irene Roberts[18,19,151], Catherine Roughley[76], Noemi Roy[18,19,151], Omid Sadeghi-Alavijeh[85], Moin Saleem[27], Nilesh Samani[152], Alba Sanchis-Juan[1,16], Ravishankar Sargur[73], Simon Satchell[27], Sinisa Savic[153], Laura Scelsi[87], Sol Schulman[154], Marie Scully[115], Claire Searle[155], Werner Seeger[86], Carrock Sewell[156], Denis Seyres[1,16], Susie Shapiro[65], Olga Sharmardina[1,16], Rakefet Shtoyerman[157], Keith Sibson[137], Lucy Side[3], Ilenia Simeoni[1,16], Michael Simpson[158], Suthesh Sivapalaratnam[55], Anne-Bine Skytte[159], Katherine Smith[45], Kenneth G C Smith[29,160], Katie Snape[161], Florent Soubrier[79], Simon Staines[1,16], Emily Staples[29], Hannah Stark[1,16], Jonathan Stephens[1,16], Kathleen Stirrups[1,16], Sophie Stock[1,16], Jay Suntharalingam[129], Emilia Swietlik[29], R Campbell Tait[162], Kate Talks[28], Rhea Tan[69], James Thaventhiran[29], Andreas Themistocleous[25], Moira Thomas[163], Kate Thomson[67,68], Adrian Thrasher[9], Chantal Thys[84], Marc Tischkowitz[164], Catherine Titterton[1,16], Cheng-Hock Toh[71], Mark Toshner[29], Matthew Traylor[69], Carmen Treacy[29,148], Richard Trembath[41], Salih Tuna[1,16], Wojciech Turek[44], Ernest Turro[1,16,17], Tom Vale[25], Chris Van Geet[84], Natalie Van Zuydam[25], Marta Vazquez-Lopez[49], Julie von Ziegenweidt[1,16], Anton Vonk Noordegraaf[30], Quintin Waisfisz[30], Suellen Walker[9], James Ware[38,39,58], Hugh Watkins[67,68,103], Christopher Watt[1,16], Andrew Webster[14,15], Wei Wei[50], Steven Welch[100], Julie Wessels[92], Sarah Westbury[145,146], John-Paul Westwood[115], John Wharton[62], Deborah Whitehorn[1,16], James Whitworth[11,12,13], Martin R Wilkins[62], Catherine Williamson[48,165], Edwin Wong[98], Nicholas Wood[166,167], Yvette Wood[1,16], Geoff Woods[11,114], Emma Woodward[77], Stephen Wort[39,41], Austen Worth[9], Katherine Yates[1,16,29], Patrick Yong[168], Tim Young[1,16], Ping Yu[1,16], Patrick Yu-Wai-Man[50]

Affiliations

[1]NIHR BioResource, Cambridge University Hospitals NHS Foundation, Cambridge Biomedical Campus, Cambridge, UK. [2]Chapel Allerton Hospital, Leeds Teaching Hospitals NHS Trust, Leeds, UK. [3]North East Thames Regional Genetics Service, Great Ormond Street Hospital for Children NHS Foundation Trust, London, UK. [4]MRC Clinical Sciences Centre, Faculty of Medicine, Imperial College London, London, UK. [5]Institute of Genetics and Molecular Medicine, University of Edinburgh, Edinburgh, UK. [6]Salford Royal NHS Foundation Trust, Salford, UK. [7]Queens Centre for Haematology and Oncology, Castle Hill Hospital, Hull and East Yorkshire NHS Trust, Cottingham, UK. [8]European Molecular Biology Laboratory, European Bioinformatics Institute (EMBL-EBI), Wellcome Genome Campus, Hinxton, Cambridge, UK. [9]Great Ormond Street Hospital for Children NHS Foundation Trust, London, UK. [10]University Hospitals Birmingham NHS Foundation Trust, Birmingham, UK. [11]Department of Medical Genetics, Cambridge Institute for Medical Research, University of Cambridge, Cambridge Biomedical Campus, Cambridge, UK. [12]Cancer Research UK Cambridge Centre, Cambridge Biomedical Campus, Cambridge, UK. [13]NIHR Cambridge Biomedical Research Centre, Cambridge Biomedical Campus, Cambridge, UK. [14]Moorfields Eye Hospital NHS Foundation Trust, London, UK. [15]UCL Institute of Ophthalmology, University College London, London, UK. [16]Department of Haematology, University of Cambridge, Cambridge Biomedical Campus, Cambridge, UK. [17]MRC Biostatistics Unit, Cambridge Institute of Public Health, University of Cambridge, Cambridge, UK. [18]MRC Molecular Haematology Unit, Weatherall Institute of Molecular Medicine, University of Oxford, Oxford, UK. [19]NIHR Oxford Biomedical Research Centre, Oxford University Hospitals Trust, Oxford, UK. [20]Center for Clinical Transfusion Medicine, University Hospital of Tübingen, Tübingen, Germany. [21]The Katharine

Dormandy Haemophilia Centre and Thrombosis Unit, Royal Free London NHS Foundation Trust, London, UK. [22]University College London, London, UK. [23]Department of Clinical Genetics, Leicester Royal Infirmary, University Hospitals of Leicester, Leicester, UK. [24]University of Leicester, Leicester, UK. [25]The Nuffield Department of Clinical Neurosciences, University of Oxford, John Radcliffe Hospital, Oxford, UK. [26]Illumina Limited, Chesterford Research Park, Little Chesterford, Nr Saffron Walden, UK. [27]Bristol Renal, University of Bristol, Bristol, UK. [28]Haematology Department, Royal Victoria Infirmary, The Newcastle upon Tyne Hospitals NHS Foundation Trust, Newcastle upon Tyne, UK. [29]Department of Medicine, School of Clinical Medicine, University of Cambridge, Cambridge Biomedical Campus, Cambridge, UK. [30]Department of Pulmonary Medicine, VU University Medical Centre, Amsterdam, The Netherlands. [31]Southampton General Hospital, University Hospital Southampton NHS Foundation Trust, Southampton, UK. [32]MRC Social, Genetic & Developmental Psychiatry Centre, Institute of Psychiatry, Psychology & Neuroscience, King's College London, London, UK. [33]NIHR Biomedical Research Centre for Mental Health, Maudsley Hospital, London, UK. [34]Newcastle University, Newcastle upon Tyne, UK. [35]Newcastle upon Tyne Hospitals NHS Foundation Trust, Newcastle upon Tyne, UK. [36]Department of Clinical Genetics, Royal Devon & Exeter Hospital, Royal Devon and Exeter NHS Foundation Trust, Exeter, UK. [37]Department of Immunology, Leicester Royal Infirmary, Leicester, UK. [38]National Heart and Lung Institute, Imperial College London, Royal Brompton Hospital, London, UK. [39]Royal Brompton Hospital, Royal Brompton and Harefield NHS Foundation Trust, London, UK. [40]Royal Free London NHS Foundation Trust, London, UK. [41]King's College London, London, UK. [42]Guy's and St Thomas' Hospital, Guy's and St Thomas' NHS Foundation Trust, London, UK. [43]King's College Hospital NHS Foundation Trust, London, UK. [44]High Performance Computing Service, University of Cambridge, Cambridge, UK. [45]Genomics England Ltd, London, UK. [46]Epidemiology and Biostatistics, Imperial College London, London, UK. [47]Imperial College Healthcare NHS Trust, London, UK. [48]Division of Women's Health, King's College London, London, UK. [49]Women’s Health Research Centre, Surgery and Cancer, Faculty of Medicine, Hammersmith Hospital, Imperial College Healthcare NHS Trust, London, UK. [50]Department of Clinical Neurosciences, School of Clinical Medicine, University of Cambridge, Cambridge Biomedical Campus, Cambridge, UK. [51]Medical Research Council Mitochondrial Biology Unit, Cambridge Biomedical Campus, Cambridge, UK. [52]Children's Renal and Urology Unit, Nottingham Children's Hospital, QMC, Nottingham University Hospitals NHS Trust, Nottingham, UK. [53]Golden Jubilee National Hospital, Glasgow, UK. [54]West Midlands Regional Genetics Service, Birmingham Women's and Children's NHS Foundation Trust, Birmingham, UK. [55]The Royal London Hospital, Barts Health NHS Foundation Trust, London, UK. [56]The Arthur Bloom Haemophilia Centre, University Hospital of Wales, Cardiff, UK. [57]Sheffield Pulmonary Vascular Disease Unit, Royal Hallamshire Hospital NHS Foundation Trust, Sheffield, UK. [58]MRC London Institute of Medical Sciences, Imperial College London, London, UK. [59]National Heart Research Institute Singapore, National Heart Centre Singapore, Singapore, Singapore. [60]Division of Cardiovascular & Metabolic Disorders, Duke-National University of Singapore, Singapore, Singapore. [61]Imperial College Renal and Transplant Centre, Hammersmith Hospital, Imperial College Healthcare NHS Trust, London, UK. [62]Department of Medicine, Imperial College London, London, UK. [63]National Pulmonary Hypertension Service (Newcastle), The Newcastle upon Tyne Hospitals NHS Foundation Trust, Newcastle upon Tyne, UK. [64]Institute of Cellular Medicine, Faculty of Medical Sciences, Newcastle University, Newcastle upon Tyne, UK. [65]Oxford Haemophilia and Thrombosis Centre, The Churchill Hospital, Oxford University Hospitals NHS Trust, Oxford, UK. [66]Department of Molecular Medicine, General Biology, and Medical Genetics Unit, University of Pavia, Pavia, Italy. [67]Department of Cardiovascular Medicine, Radcliffe Department of

Medicine, University of Oxford, Oxford, UK. [68]Oxford University Hospitals NHS Foundation Trust, Oxford, UK. [69]Stroke Research Group, Department of Clinical Neurosciences, University of Cambridge, Cambridge Biomedical Campus, Cambridge, UK. [70]Nottingham University Hospitals NHS Trust, Nottingham, UK. [71]The Roald Dahl Haemostasis and Thrombosis Centre, The Royal Liverpool Hospital, Liverpool, UK. [72]Regional Immunology Service, Kelvin Building, Royal Victoria Hospital, Belfast, UK. [73]Sheffield Teaching Hospitals NHS Foundation Trust, Sheffield, UK. [74]Pathology and Laboratory Medicine, University of Western Australia, Crawley, Australia. [75]Ramón Sardá Mother's and Children's Hospital, Buenos Aires, Argentina. [76]Haemophilia Centre, Kent & Canterbury Hospital, East Kent Hospitals University Foundation Trust, Canterbury, UK. [77]Manchester Centre for Genomic Medicine, Saint Mary's Hospital, Manchester, UK. [78]Salisbury District Hospital, Salisbury NHS Foundation Trust, Salisbury, UK. [79]Departement de genetique, Hopital Pitie-Salpetriere, Paris, France. [80]Service d'Hématologie Biologique, Hôpital d'enfants Armand Trousseau, Paris, France, Paris, France. [81]Inserm U1170, Villejuif, France. [82]Assistance Publique-Hôpitaux de Paris, Département d'Hématologie, Hôpital Armand Trousseau, Paris, France. [83]Department of Paediatrics, School of Clinical Medicine, University of Cambridge , Cambridge Biomedical Campus, Cambridge, UK. [84]Department of Cardiovascular Sciences, Center for Molecular and Vascular Biology, University of Leuven, Leuven, Belgium. [85]UCL Centre for Nephrology, University College London, London, UK. [86]University of Giessen and Marburg Lung Center (UGMLC), Giessen, Germany. [87]Division of Cardiology, Fondazione IRCCS Policlinico S. Matteo, Pavia, Italy. [88]UCL Great Ormond Street Institute of Child Health, London, UK. [89]Universite Paris-Sud, Le Kremlin-Bicêtre, France. [90]Service de Pneumologie, DHU Thorax Innovation, Hôpital Bicêtre, Le Kremlin-Bicêtre, France. [91]INSERM U999, LabEx LERMIT, Centre Chirurgical Marie Lannelongue, Le Plessis Robinson, France. [92]University Hospitals of North Midlands NHS Trust, Stoke-on-Trent, UK. [93]East Yorkshire Regional Adult Immunology and Allergy Unit, Hull Royal Infirmary, Hull & East Yorkshire Hospitals NHS Trust, Hull, UK. [94]Department of Clinical Genetics, Liverpool Women's NHS Foundation, Liverpool, UK. [95]Institute for Immunology and Transfusion Medicine, University of Greifswald, Greifswald, Germany. [96]Section of Internal and Cardiovascular Medicine, University of Perugia, Perugia, Italy. [97]Mitochondrial Research Group, Institute of Genetic Medicine, Newcastle University, Newcastle upon Tyne, UK. [98]Institute of Genetic Medicine, Newcastle University, Newcastle upon Tyne, UK. [99]Barts Health NHS Foundation Trust, London, UK. [100]Birmingham Heartlands Hospital, Heart of England NHS Foundation Trust, Birmingham, UK. [101]ARCH: Australian Research Centre for Health of Women and Babies, Robinson Research Institute, Discipline of Obstetrics and Gynaecology, The University of Adelaide, Women's and Children's Hospital, Adelaide, Australia. [102]Department of Neurology, Sheffield Teaching Hospitals NHS Foundation Trust, Sheffield, UK. [103]Wellcome Trust Centre for Human Genetics, University of Oxford, Oxford, UK. [104]Department of Neurology, Leeds Teaching Hospital NHS Trust, Leeds, UK. [105]Epsom & St Helier University Hospitals NHS Trust, London, UK. [106]Northern Genetics Service, The Newcastle upon Tyne Hospitals NHS Foundation Trust, International Centre for Life, Newcastle upon Tyne, UK. [107]Wellcome Centre for Mitochondrial Research, Institute of Genetic Medicine, Newcastle University, Newcastle upon Tyne, UK. [108]John Walton Muscular Dystrophy Research Centre, Institute of Genetic Medicine, Newcastle University, Newcastle upon Tyne, UK. [109]Wellcome Trust Sanger Institute, Hinxton, Cambridge, UK. [110]Department of Clinical Genetics, Guy's and St Thomas' NHS Foundation Trust, London, UK. [111]Department of Paediatric Nephrology, Great North Children's Hospital, Newcastle upon Tyne Hospitals NHS Foundation Trust, Newcastle upon Tyne, UK. [112]University Hospital of Wales, Cardiff, UK. [113]Cardiff & Vale University LHB, Cardiff, UK. [114]Addenbrookes Hospital, Cambridge University Hospitals NHS Foundation Trust, Cambridge,

UK. [115]University College London Hospitals NHS Foundation Trust, London, UK. [116]Ludwig Boltzmann Institute for Lung Vascular Research, Graz, Austria. [117]Dept of Internal Medicine, Division of Pulmonology, Medical University of Graz, Graz, Austria. [118]Division of Transplantation Immunology and Mucosal Biology, Department of Experimental Immunobiology, Faculty of Life Sciences and Medicine, King's College London, London, UK. [119]Department of Paediatric Nephrology, Evelina London Children's Hospital, Guy's & St Thomas' NHS Foundation Trust, London, UK. [120]Department of Pediatric Hematology, Immunology, Rheumatology and Infectious Diseases, Emma Children's Hospital, Academic Medical Center (AMC), University of Amsterdam, Amsterdam, The Netherlands. [121]Department of Clinical Genetics, Academic Medical Center (AMC), University of Amsterdam, Amsterdam, The Netherlands. [122]Molecular Neurosciences, Developmental Neurosciences, UCL Great Ormond Street Institute of Child Health, London, UK. [123]Department of Neurology, Great Ormond Street Hospital for Children NHS Foundation Trust, London, UK. [124]Department of Haematology, Hammersmith Hospital, Imperial College Healthcare NHS Trust, London, UK. [125]Department of Haematology, Imperial College London, London, UK. [126]Division of Hematology, The Children's Hospital of Philadelphia, Philadelphia, USA. [127]Department of Pediatrics, Perelman School of Medicine at the University of Pennsylvania, Philadelphia, USA. [128]Department of Infection, Immunity & Cardiovascular Disease, University of Sheffield, Sheffield, UK. [129]Royal United Hospitals Bath NHS Foundation Trust, Bath, UK. [130]Department of Haematology, Guy's and St Thomas' NHS Foundation Trust, London, UK. [131]Cambridge NIHR Biomedical Research Centre, Cambridge Biomedical Campus, Cambridge, UK. [132]Haemophilia, Haemostasis and Thrombosis Centre, Hampshire Hospitals NHS Foundation Trust, Basingstoke, UK. [133]Wallenberg Laboratory, Department of Molecular and Clinical Medicine, Sahlgrenska Academy, University of Gothenburg, Gothenburg, Sweden. [134]Faculty of Medical and Human Sciences, Centre for Endocrinology and Diabetes, Institute of Human Development, University of Manchester, Manchester, UK. [135]Department of Clinical Neurophysiology, Manchester Royal Infirmary, Central Manchester University Hospitals National Health Service Foundation Trust, Manchester Academic Health Science Centre, Manchester, UK. [136]National Institute for Health Research/Wellcome Trust Clinical Research Facility, Manchester, UK. [137]Department of Haematology, Great Ormond Street Hospital for Children NHS Foundation Trust, London, UK. [138]The National Hospital for Neurology and Neurosurgery, University College London Hospitals NHS Foundation Trust, London, UK. [139]Royal Hospital for Children, NHS Greater Glasgow and Clyde, Glasgow, UK. [140]Oxford Centre for Diabetes, Endocrinology and Metabolism, University of Oxford, Churchill Hospital, Oxford, UK. [141]Centre for Haematology, Department of Medicine, Hammersmith Hospital, Imperial College Healthcare NHS Trust, London, UK. [142]Department of Clinical Genetics, Addenbrookes Hospital, Cambridge University Hospitals NHS Foundation Trust, Cambridge, UK. [143]MPGN/C3 Glomerulopathy Rare Renal Disease group, , UK. [144]Institute of Neuroscience and Psychology, University of Glasgow, Glasgow, UK. [145]School of Cellular and Molecular Medicine, University of Bristol, Bristol, UK. [146]University Hospitals Bristol NHS Foundation Trust, Bristol, UK. [147]East Anglian Regional Genetics Service, Cambridge University Hospitals NHS Foundation Trust, Cambridge, UK. [148]Royal Papworth Hospital NHS Foundation Trust, Cambridge, UK. [149]Pain Research, Department of Surgery and Cancer, Faculty of Medicine, Imperial College London, London, UK. [150]Chelsea and Westminster Hospital NHS Foundation Trust, London, UK. [151]Department of Paediatrics, Weatherall Institute of Molecular Medicine, University of Oxford, Oxford, UK. [152]Departments of Cardiovascular Sciences and NIHR Leicester Cardiovascular Biomedical Research Unit, University of Leicester, Leicester, UK. [153]The Leeds Teaching Hospitals NHS Trust, Leeds, UK. [154]Beth Israel Deaconess Medical Centre and Harvard Medical School, Boston, USA. [155]Department of

Clinical Genetics, Nottingham University Hospitals NHS Trust, Nottingham, UK. [156]Scunthorpe General Hospital, Northern Lincolnshire and Goole NHS Foundation Trust, Scunthorpe, UK. [157]Clinical Genetics Institute, Kaplan Medical Center, Rehovot, Israel. [158]Genetics and Molecular Medicine, King's College London, London, UK. [159]Aarhus University Hospital, Aarhus, Denmark. [160]Cambridge Institute for Medical Research, University of Cambridge, Cambridge Biomedical Campus, Cambridge, UK. [161]Department of Clinical Genetics, St George's University Hospitals NHS Foundation Trust, London, UK. [162]Glasgow Royal Infirmary, NHS Greater Glasgow and Clyde, Glasgow, UK. [163]Gartnavel General Hospital, NHS Greater Glasgow and Clyde, Glasgow, UK. [164]Addenbrooke's Treatment Centre, Addenbrooke's Hospital, Cambridge University Hospitals NHS Foundation Trust, Cambridge, UK. [165]Institute of Reproductive and Developmental Biology, Surgery and Cancer, Hammersmith Hospital, Imperial College Healthcare NHS Trust, London, UK. [166]Department of Molecular Neuroscience, UCL Institute of Neurology, London, UK. [167]UCL Genetics Institute, London, UK. [168]Frimley Park Hospital, NHS Frimley Health Foundation Trust, Camberley, UK.

US PAH Biobank Consortium

Alison Theuer[72], Anagha Malur[59], Ann Williams[36], Anne Dotson[73], Ashley Warden[18], Brandy Harrington[69], Brenda Vang[69], Caitlin Ziemak[22], Nancy Casanova[41], Elizabeth Caskey[71], Catherine MacDonald[41], Courtney Rowley[18], Daniel Larimore[18], Daniela Brady[63], David Tomer[35], Anne Davis[75], Debra Broach[50], Jane Devereux[63], Ellen Lovato[27], Eric Stratton[66], Erin Turk[50], Esperanza Jackson[21], Gina Paciotti[69], Gretchen Peichel[69], Hellina Birru[22], Holly del Junco[20], Rebecca Ingledue[36], Jackie Bruno[72], Jan Drake[24], Jennifer Marks[50], Jessica Pisarcik[67], Jill Spears[18], Joseph Santiago[24], Jordyn DeMartino[18], Joy Beckmann[57], Julia Palmer[18], Karen Visnaw[44], Karla Kennedy[37], Karlise Lewis[46], Kathleen Miller-Reed[46], Kelly Hannon[20], Kimberly McClain[73], Laura Dillon[69], Lekan Olanipekun[70], Lillian Mendibles[41], Lindsey Hawke[17], Linnea Brody[75], Louise Durst[39], Mary Andrews[65], Melissa Allahua[47], Melissa Stratoberdha[26], Merte Lemma[56], Molly Cope[73], Mya Franzo[73], Natalia Feliz[21], Nicholas Hawkes[41], Tracy Norwood[71], Opal Wilson[71], Amy Palmisciano[47], Peggy Gruhlke[39], Priscilla Correa[23], Randy Blake[57], Reema Karnekar[59], Robyn Do[18], Kristal Rohwer[39], Sara Ahmed[22], Kimberly Schiltz[15], Page Scovel[15], Shannon Cordell[60], Sharon Heuerman[27], Shazzra Ahmad[64], Sylwia Szuberla[20], Tammy Roads[36], Thoeun Iem[39], Traci Mcgaha[67], Tracy Urban[46], Valerie Aston[35], Waleed Ayesh[64], Allison Light[72], Abby Arkon[19], Andrea Tavlarides[26], Audrey Anderson[21], John Bindu[57], Deedre Boekwig[35], Donna Singleton[71], Inna Abrea[26], Jennifer Lee[40], Mark Ormiston[17], Renesa Whitman[21], Royanne Holy[40], Sisama Almeida-Peters[37], Tosin Igenoza[70], Hap Farber[66], Auvo Reponen[45], Mukta Barve[45], Amber Gygi[45], Clayborne Winslow[45]

[9] Division of Human Genetics, Cincinnati Children's Hospital Medical Center, Department of Pediatrics, University of Cincinnati College of Medicine, Cincinnati, OH, United States;
[15] Division of Cardiovascular Medicine, University of Iowa, Iowa City IA, United States;
[17] Queen’s University, Kingston ON, Canada;
[18] Medical University of South Carolina, Charleston SC, United States;
[19] Vanderbilt University-Peds, Nashville TN, United States;
[20] University of Colorado Denver, Aurora CO, United States;

[21] Baylor Research Institute, Plano TX, United States;
[22] Medstar Health, Washington D.C., United States;
[23] Allegheny-Singer Research Institute, Pittsburgh PA, United States;
[24] Ohio State University, Columbus OH, United States;
[26] Mayo Clinic Florida, Jacksonville FL, United States;
[27] Washington University, St. Louis MO, United States;
[35] Department of Medicine at Intermountain Medical Center and the University of Utah, Murray UT, United States;
[36] University of Cincinnati, Cincinnati OH, United States;
[37] Duke University Medical Center, Durham NC, United States;
[39] Mayo Clinic, Rochester MN, United States;
[40] Weill Cornell Medical College and The Houston Methodist Hospital, Houston TX, United States;
[41] Department of Medicine and Arizona Health Sciences Center, University of Arizona, Tucson, AZ, United States;
[44] Tufts Medical Center, Boston MA, United States;
[45] Cincinnati Children’s Hospital, Cincinnati OH, United States;
[46] Children’s Hospital of Colorado, University of Colorado Denver, Aurora CO, United States;
[47] Rhode Island Hospital, Providence RI, United States;
[50] Indiana University, Indianapolis IN, United States;
[56] Inova Heart and Vascular Institute, Falls Church VA, United States;
[57] LA Biomedical Research Institute at Harbor-UCLA, Torrance CA, United States;
[59] East Carolina University, Greenville NC, United States;
[60] Vanderbilt University Medical Center, Nashville TN, United States;
[63] Columbia University, New York NY, United States;
[64] Wayne State University, Detroit MI, United States;
[65] University Hospital of Cleveland, Cleveland OH, United States;
[66] Boston University School of Medicine, Boston MA, United States;
[67] University of Pittsburgh, Pittsburgh PA, United States;
[69] University of Minnesota, Minneapolis MN, United States;
[70] UT Southwestern, Dallas TX, United States;
[71] LSU Health, Shreveport LA, United States;
[72] University of Rochester Medical Center, Rochester NY, United States;
[73] Spectrum Health Hospitals, Grand Rapids MI, United States;
[75] Seattle Children's Hospital, Seattle WA, United States;
