# Supplementary material for: Mendelian randomisation analysis of red cell distribution width in pulmonary arterial hypertension
Source: Eur Respir J. 2020 Feb 13;55(2):1901486. doi: 10.1183/13993003.01486-2019 (PMC7015630; doi:10.1183/13993003.01486-2019)
Supplement: Supplementary file 2 [file ERJ-01486-2019.Shareable.pdf]

SHAREABLE PDF

Mendelian randomisation analysis of red cell distribution width in pulmonary arterial hypertension

Anna Ulrich[1], John Wharton[1], Timothy E. Thayer[2], Emilia M. Swietlik[3,4], Tufik R. Assad[5], Ankit A. Desai[6], Stefan Gräf[3,7,8], Lars Harbaum[1], Marc Humbert[9,10,11], Nicholas W. Morrell[3,7], William C. Nichols[12], Florent Soubrier[13], Laura Southgate[14], David-Alexandre Trégouët[15], Richard C. Trembath[16], Evan L. Brittain[2,17], Martin R. Wilkins[1], Inga Prokopenko[18,19,21] and Christopher J. Rhodes[1,21], on behalf of The NIHR BioResource – Rare Diseases Consortium[20], the UK PAH Cohort Study Consortium[20] and the US PAH Biobank Consortium[20]

Affiliations: [1]National Heart and Lung Institute, Hammersmith Campus, Imperial College London, London, UK. [2]Vanderbilt University Medical Center, Division of Cardiovascular Medicine, Nashville, TN, USA. [3]Dept of Medicine, University of Cambridge, Cambridge, UK. [4]Pulmonary Vascular Disease Unit, Royal Papworth Hospital NHS Foundation Trust, Cambridge, UK. [5]Williamson Country Medical Center, Franklin, TN, USA. [6]Dept of Medicine, Indiana University, Indianapolis, IN, USA. [7]NIHR BioResource – Rare Diseases, Cambridge, UK. [8]Dept of Haematology, University of Cambridge, Cambridge, UK. [9]Université Paris-Sud, Faculté de Médecine, Université Paris-Saclay, Paris, France. [10]AP-HP, Service de Pneumologie, Centre de référence de l'hypertension pulmonaire, Hôpital Bicêtre, Le Kremlin-Bicêtre, France. [11]INSERM UMR_S 999, Hôpital Marie Lannelongue, Le Plessis Robinson, France. [12]Division of Human Genetics, Cincinnati Children's Hospital Medical Center, Dept of Pediatrics, University of Cincinnati College of Medicine, Cincinnati, OH, USA. [13]Sorbonne Universités, UPMC Univ. Paris 06, Institut National pour la Santé et la Recherche Médicale (INSERM), Unité Mixte de Recherche en Santé (UMR_S) 1166, Paris, France. [14]Molecular and Clinical Sciences Research Institute, St George's University of London, London, UK. [15]INSERM UMR_S 1219, Bordeaux Population Health Research Center, University of Bordeaux, Bordeaux, France. [16]Division of Genetics and Molecular Medicine, King's College London, London, UK. [17]Vanderbilt Translational and Clinical Cardiovascular Research Center, Nashville, TN, USA. [18]Dept of Clinical and Experimental Medicine, University of Surrey, Guildford, UK. [19]Dept of Metabolism, Digestion and Reproduction, Imperial College London, London, UK. [20]Members listed in the supplementary material. [21]These authors contributed equally.

Correspondence: Christopher J. Rhodes, National Heart and Lung Institute, Medicine, Imperial College London, London, W12 0NN, UK. E-mail: crhodes@imperial.ac.uk

@ERSpublications
Mendelian randomisation using genetic data from the largest-to-date PAH cohort does not support red cell distribution width or iron deficiency as a cause of PAH, which is important when interpreting iron replacement trials in this condition http://bit.ly/2PPaa88

Cite this article as: Ulrich A, Wharton J, Thayer TE, *et al.* Mendelian randomisation analysis of red cell distribution width in pulmonary arterial hypertension. *Eur Respir J* 2020; 55: 1901486 [https://doi.org/10.1183/13993003.01486-2019].

This single-page version can be shared freely online.

ABSTRACT Pulmonary arterial hypertension (PAH) is a rare disease that leads to premature death from right heart failure. It is strongly associated with elevated red cell distribution width (RDW), a correlate of several iron status biomarkers. High RDW values can signal early-stage iron deficiency or iron deficiency anaemia. This study investigated whether elevated RDW is causally associated with PAH.

A two-sample Mendelian randomisation (MR) approach was applied to investigate whether genetic predisposition to higher levels of RDW increases the odds of developing PAH. Primary and secondary MR

Copyright ©ERS 2020. This version is distributed under the terms of the Creative Commons Attribution Licence 4.0.

analyses were performed using all available genome-wide significant RDW variants (n=179) and five genome-wide significant RDW variants that act *via* systemic iron status, respectively.

We confirmed the observed association between RDW and PAH (OR 1.90, 95% CI 1.80–2.01) in a multicentre case–control study (cases n=642, disease controls n=15 889). The primary MR analysis was adequately powered to detect a causal effect (odds ratio) between 1.25 and 1.52 or greater based on estimates reported in the RDW genome-wide association study or from our own data. There was no evidence for a causal association between RDW and PAH in either the primary (OR_{causal} 1.07, 95% CI 0.92–1.24) or the secondary (OR_{causal} 1.09, 95% CI 0.77–1.54) MR analysis.

The results suggest that at least some of the observed association of RDW with PAH is secondary to disease progression. Results of iron therapeutic trials in PAH should be interpreted with caution, as any improvements observed may not be mechanistically linked to the development of PAH.
